# Emerging analytical techniques for lipid profiling in food products: insights into processing effects and quality control

**DOI:** 10.1016/j.fochx.2025.103360

**Published:** 2025-12-03

**Authors:** Deepika Kathuria, Sonal Aggarwal, Akanksha Negi, Riya Barthwal, Aroma Joshi, Narpinder Singh

**Affiliations:** Department of Food Science and Technology, Graphic Era (deemed to be University), Dehradun 248002, Uttarakhand, India

**Keywords:** Lipids, food, mass-spectrometry, food processing, metabolomic

## Abstract

Lipids are essential components of foods which include fatty acids, glycolipids, sphingolipids, sterols, vitamin D, isoprenoids, etc. It possesses bioactive properties that exhibit desirable impact on human health. However, understanding the complexity of food lipids, requires sophisticated analytical methods capable of capturing their full spectrum. Lipidomics (LIP), an emerging omics field derived from metabolomics focuses on the complete analysis of lipid molecules within food matrices. Mass spectrometry (MS)-based LIP, exhibit great potential for analysing food lipids. The present review discusses the application of LIP in characterizing lipid metabolites in food, including how food processing, cooking, and storage, influence lipid profiles. Advancement in MS-based LIP enabled the detection of lipid alterations, along with ensuring food quality, authenticity, and safety. Future trends suggest further advancements in MS and separation techniques and application of Large Language Models will emerge as powerful tools in supporting oil processing research and industry practices. These techniques offered innovative predictions for mining significant information from lipidomic related large volumes of scientific and technical literature that will expand the application of LIP, providing deeper insights into lipid metabolism as well as understanding lipids in relation to health and nutrition.

## Highlights

• Food lipid include fatty acids, glycolipids, sphingolipids, sterols, etc
• Lipidomics involve application of advanced mass spectrometry techniques
• Provide insights into food lipid complexity, lipid oxidation during processing
• Advance AI-powdered system relates oil processing parameters to quality metrics

## 1 Introduction

Lipids, an essential component in human diet plays multiple roles in maintaining health. They include a range of hydrophobic and amphiphilic molecules that act as a source of energy, essential nutrients, and key elements in biological processes. Several lipids, such as essential fatty acids (FAs) and fat-soluble vitamins (e.g., vitamins A, D, E, and K), are not produced by the human body and has to be acquired from diet (Custers et al., 2022). Besides nutritional value, these lipids also contribute in retaining the structural and functional integrity of cells, being involved in critical biological functions like membrane formation, signal transduction, and energy storage (Yu et al., 2021). Moreover, abnormal lipid metabolism led to occurrence of chronic diseases like cardiovascular diseases, neurodegenerative disease, inflammatory reaction, and cancer (Xu et al., 2024). Beyond their biological roles, in food, lipids act as a fundamental component in providing desirable colour, flavour, texture and mouthfeel. During processing and storage of food, lipids undergo oxidative and hydrolytic reaction, that exhibit both desirable and undesirable impact on end food product quality. Monitoring lipid composition and alteration in lipid structures during these stages is necessary for upholding food quality and ensuring safety. In recent years, LIP, a metabolomics field has appeared as an important tool for the comprehensive analysis of lipid molecules in food matrices. LIP allows characterization of 100s to 1000s of lipid species in complex food samples. The application of LIP in food science, often referred to as food LIP, has rapidly advanced in recent years. Food LIP played essential role in understanding the nutritional and functional properties of lipids in different food products, evaluate food processing methods, and assess the lipid oxidation reaction taken place in food products that may affect their safety and shelf life. Additionally, it also ensures food authenticity, traceability, and quality by detecting food adulteration. Various physical methods such as gas chromatography (GC), mass spectrometry (MS), and near-infrared spectrometry (NIS) are frequently employed to analyse the FA composition in raw foods products altered by varying growing condition, type of fed consumed, etc., development of undesirable flavours, the formation of volatile compounds, alterations in FA composition, and degradation of phenolic compounds due to occurrence of oxidative reaction in vegetable oils. However, the complexity and diversity of the food lipidome are quite challenging, and one single methodological approach cannot address the global lipidome; thus, specific approaches are needed to address the challenges of the plethora of lipids found in food. In recent years, new technologies have emerged to tackle these challenges. High-resolution mass spectrometry techniques, such as matrix-assisted laser desorption/ionization time-of-flight (MALDI-TOF), MALDI-MS, MALDI-MSI, Quardruple-TOF (QTOF), and Trapped Ion Mobility Spectrometry TOF (TIMS-QTOF), Electrospray Ionization MS (ESI-MS) and ESI-MS/MS often combined with chromatographic methods and multivariate statistical analysis, have been increasingly utilized in LIP to investigate the fundamental chemical properties of lipids. These advanced tools enable the detection and quantification of both high and low level lipids with high precision (Martakos et al., 2024, Zhou et al., 2021; Wood et al., 2021). This MS-based LIP technique has revolutionized food lipid analysis, enabling the detailed examination of lipid profiles, oxidation processes, and bioactive lipid components. Hence, the present review provides an in-depth overview of food LIP, focusing on the diverse classes of lipids in food and the MS-based techniques used for their analysis. It highlights the unique challenges of characterizing complex lipidomes in different food matrices and the critical importance of LIP in modern food science. Additionally, it addresses how lipid composition changes during processing and storage, the analytical challenges of lipid profiling, and future trends in this rapidly growing research area. By advancing the field of food LIP, researchers aim to enhance the health potential properties of dietary lipids and ensure food quality and safety.

## 2 Lipids in food

Lipids in food encompass a diverse range of compounds that are critical for both nutrition and food quality. Based on the LipidMaps classification, lipids are grouped into categories such as fatty acyls, complex lipid, sterols, terpenes, and oxidized lipids. Table 1 outlines the impacts of dietary lipid and their associated health implications. Fatty acyl including FA are the carbon chain with carboxylic acid group which are further classified on the basis of chain length. Short chain FA (SCFA with C-atom < 6), medium-chain FA (MCFA with 6 to 14 C-atoms), long-chain FA (LCFA with 14 to 21 C-atoms), and very long-chain FA (VLCFA with C-atoms >22), depending on the length of the acyl chain. SCFA are found in butterfat, dairy products from cows and goats, contributing to their odour and volatility. MCFA, abundant in coconut oil [47% lauric acid (C12:0) and 19.1% myristic acid (C14:0)], which is widely used in parenteral nutrition as a rapid, accessible energy source (Furuta et al., 2023). These FA are further categorised depending upon the presence of double bond i.e., saturated (no double bond) and unsaturated (presence of double bond) where, unsaturated as classified as monounsaturated FA (MUFA) exhibiting single double bond and polyunsaturated FA (PUFA) exhibiting more than two or more double bond (Lee-Okada et al., 2025). The WHO recommends limiting intake of saturated fat to 10% of total calories. Further, the position of the double bond are also used to categorise MUFA and PUFA, such as omega 3, 6, or 9 (Lee-Okada et al., 2025). FA are vital for health, particularly PUFA, such as omega-3 and omega-6, which the body cannot synthesize. Omega FA such as docosahexaenoic acid (DHA; C22) and nervonic acid (C24) primarily obtained from fish and fish oil, are examples of unsaturated very long-chain FA (VLCFA). Saturated versions occur in low amounts (less than 1%) in vegetable oils, except peanut oil, which contains high content of lignoceric (C24; 1.39%) and behenic (C22; 2.62%) acids (Fabiszewska & Białecka-Florjańczyk, 2021). The oxidation of unsaturated FA (UFA) also produces aromatic molecules that enhance food flavours, such as those derived from linoleic and linolenic acids. Besides FA, certain waxes, fatty esters are also included in the fatty acyl category which are derived from cuticular wax, the exterior-most layer that wraps the surface of plant organs such as fruits and leaves. Additionally, cuticular wax also helps fruit in reducing mechanical damage, thereby maintaining its commodity value (Wu et al., 2023). Waxes impart texture of foods, helping conserving firmness and sensory qualities. Additionally, edible films and coatings crafted from waxes like candelilla and carnauba are used to extend the freshness of fruits, including fresh-cut ones (de Freitas et al., 2019). Innovative wax-based oleogels have also been proposed as a low-calorie fat alternative (Gengatharan et al., 2023). (See Table 2.)

**Table 1:** Lipids in food

**Broader category of lipids**	**Type of lipid**	**Example of lipid**	**Source of lipid**	**Health Implications**	**References**
Fatty acyls	SFA	Palm oil	Palm oil plantation	• Influences CVD	Han and He (2021)
	PUFA	DHA and EPA	Blue foods	• Positive impact on brain and heart functioning • Alleviate pathological conditions	von Gerichten et al. (2024)
		Conjugated linoleic acids	Dairy and meat products	• Positive impact against obesity • Anti-tumour properties • Adverse impact on spleen and liver	Ali et al. (2022); Deng et al. (2022)
	BCFA	Phytanic acid and Pristanic acid	Dairy and meat products	• Anti-tumour properties	Fabiszewska and Białecka-Florjańczyk (2021)
		4,8,12-Trimethyltridecanoic acid	Blue foods	-	Zhang et al. (2024)
	MUFA/PUFA	Phospholipids	Blue foods and animal sources	• Anti-inflammatory	Lordan and Wang (2020)
Complex lipids	N-acyl-phospholipids	N-acyl-phosphatidylethanolamine, and N-acyl-phosphatidylserine	Olive oil, olive seeds and lupin seeds	• Normalize appetite	Tietel et al. (2023); Calvano et al. (2020)
	Polar lipids	Sphingolipids	Dairy products and marine echinoderms	• Anti-inflammatory • Effective against Colon cancer • Regulates cell cycle	Ali et al. (2022); Deng et al. (2022)
	Glycerolipids	Glycolipids	Algae	• Effective against cancer and chronic inflammatory issue • Anti-microbial activity	Guo and Wang (2022)
		Betaine lipids	Algae (*Ulva* and *Chlamydomonas*), olive oil and olives	• *Still unknown*	Jouhet et al. (2024); Guo and Wang (2022)
		Triglycerides	Olive oil, marine, flaxseeds	• Source of energy	Jardim et al. (2024); Zhang et al. (2021)
Neutral lipids	Sterols	Acylsterolglycoside	Rice, olive seeds, green tea	• Decreases cholesterol levels • Lowers the risk of CVD	Alves et al. (2021); Huang *et al*. (2018); Zhang et al. (2021)
		Ergosterol	Mushrooms	• Pro-Vitamin D	Zheng et al. (2025)
Isoprenoid	Terpenes		Fruits, herbs and spices	• Anti-diabetic and anti-inflammatory	Zhou and Pichersky (2020); Li, Howell, Fang, and Zhang (2020); Sánchez-Martínez et al. (2021)
Oxidized lipids and oxylipins	Oxidized lipids	Dietary oxidized-lipids		• Leads to inflammatory dis-orders, tumours • Transported to body tissues	Kuksis and Pruzanski (2023)
		Phytosterol (90 μg/g)	French fries, spreads, crisp	• Reduces cholesterol	Borel et al. (2023)

**Table 2:** Overview on lipidomic technique

**Technique**	**Equipment**	**Principle**	**Merits**	**Demerits**	**Reference**
APCI-MS	APCI-LC/MS	Ionize lipids in a liquid sample by exposing them to a heated atmosphere under atmospheric pressure	Operates at atmospheric pressure, allowing for direct ionization of samples from the liquid phase without the need for vacuum systems Less affected by matrix interference, Robust, Handle wide range of samples	Cannot ionize easily the hydrocarbon waxes	Saliu (2022)
DESI-MSI	DESI-IM-QTOF MS	Uses fine spray of charged solvent droplets to extract and ionize lipids directly from the surface of a solid sample	Ionize the tissue samples directly without any pre-processing Analysis without the need for extensive sample preparation, high spatial resolution	Low quality of the observed MS/MS fragmentation processes.	Hou et al. (2022)
IMS-MS	QTRAP	Separates ions based on their shape and size as they travel through an electric field	Separates ions based on their mobility in an electric field, which is influenced by their size, shape, and charge. Provides an additional dimension of separation based on ion size and shape, high-resolution separation	Complex and expensive; cannot readily separate and identify enantiomers.	Dubland (2022)
LAESI-MSI	LAESI-MS/MS	Uses laser ablation to create aerosolized particles from a solid lipid sample, which are then ionized by electrospray to facilitate mass analysis	Excites the OH vibrations in water molecules with a focused mid-IR laser beam in biological samples, suitable for samples containing an appropriate amount of water, takes ≤2 seconds time per sample	Laser can cause thermal damage to the sample; problematic while using for solid samples	Liu, Zhang, et al., 2024; Hieta *et al.* (2020)
MALDI-MSI	MALDI-TOF MS	Uses matrix material to absorb laser energy, facilitating the desorption and ionization of lipid molecules from a solid sample surface, allowing for the spatial mapping of lipid distributions and identification based on their mass-to-charge ratios	Soft ionization method, determine the spatial distribution and relative abundance of lipids, Easy operation, highly sensitive, ability to ionize a wide variety of molecules, minimizes fragmentation of the analytes,	Introduces unfavourable heterogeneity to the imaging signals	Wang, Zheng, et al., 2024
UPLC-ESI-MSI	UPLC-ESI-MS/MS and Orbitrap Exploris GC	Liquid ionization of samples by high voltage to create charged droplets that evaporate, producing gas-phase ions for mass analysis	Used for complex lipid profiling, produces intact or pseudo-molecular gas-phase ions from molecules Highly sensitive, capable of detecting low-quantity of lipids, suitable for liquid samples	May not capture all lipid-aroma interactions due to matrix complexity.	Liu et al. (2022)
UPLC-Q-Exactive Orbitrap MS	MS-DIAL, triple quadrupole MS	Uses chemical ionization at atmospheric pressure to achieve efficient gas-phase ionization of analytes.	Extremely high resolution, high mass accuracy, elucidate the specific lipid molecular species	Expensive, effective sample preparation is essential	Li et al. (2023); Hu et al. (2022)

Complex lipids are another category which consist of FA in combination with other molecular components such as sugar, alcohol, phosphate group. Key type of complex lipid includes, phospholipid (PL), glycolipid (GL), sphingolipid (SL), betaine lipid, triacylglyceride (TAG). Glycerophospholipids (GPL) are complex lipids with a backbone of glycerol including two FA, and a polar head group of phosphate. A key subclass, lyso-phospholipids, contains only one FA. PL are abundant in meat and fish, with lower levels in oils. It serves as vital sources of PUFA, especially from marine sources and are more bioavailable than neutral lipids (Canelli et al., 2022). They offer health benefits like reducing cardiovascular disease risk and possess antioxidant and anti-inflammatory properties (Lordan et al., 2020). Glycerol-based glycolipids, such as monoglycosyl diacylglycerols (MGDG), diglycosyl diacylglycerols (DGDG), and sulfoquinovosyl diacylglycerols (SQDG) are found in plant and algae. They help organisms to adjust in varied environmental conditions (Guo & Wang, 2022). MGDGs and DGDGs are neutral GL with one and two galactose molecules in their polar heads, respectively. SQDGs are sulfur-containing acidic lipids with an esterified sulfoquinovosyl group in their polar heads. Betaine lipids are non-phosphorus zwitter ionic polar lipids featuring a betaine group in the polar head and two FA esterified at first and second locations of the glycerol backbone. The three categories of betaine lipids i.e., diacylglyceryl trimethyl homoserine (DGTS), diacylglyceryl hydroxymethyl trimethyl alanine (DGTA), and diacylglyceryl carboxy hydroxymethyl choline (DGCC) are based on the structure of the polar group. While olives and olive oil contain betaine lipids (Alves et al., 2019), however primarily found in algae (Rey et al., 2022) rather than in higher plants. TAG, another important non-polar GL. It has single glycerol molecule esterified to three FA, serve as primary energy storage in plants and animals. The FA composition of TAG strongly relies on the food origin, for example, triolein is the predominant in olive oil, while TAG consisting of n-3 fatty acids are existence in foods of marine origin or flaxseed. SL are polar lipids characterized by a sphingoid base linked to fatty acyl chains, such as ceramides (Cer), or to polar head groups like phosphocholine (PC) in sphingomyelin (SM). SM is the primary SP in animal foods but is scarce in higher plants (Mamode Cassim et al., 2020). Glycosylceramides, including hexosylceramides (HexCer), are found in various starch-, water-, and oil-rich foods (Ali et al., 2022). Gangliosides have been shown to reduce gut inflammation, boost immunity, and prevent infections (Yamashita et al., 2021).

Sterols classified as neutral lipids, including plant sterols and cholesterol, were reported to be vital for managing cholesterol levels and decreasing the risk of atherosclerosis and CVD (Boren et al., 2020). Plant oils, such as those from the Brassicaceae family (rich in brassicasterol), and oils from pumpkin seeds, which contain specific sterols, are of interest for their health benefits and as indicators of food authenticity. Compared with vegetable oils, phytosterols in margarine (natural or added) are resistant to oxidation. β-Sitosterol is about 20% less prone to oxidation than campesterol. The formation and composition of phytosterol oxidation products (POPs) depend mainly on heating time, temperature, and initial phytosterol content (Xu et al., 2022). Further, terpenes, belonging to class of isopernoids that encompass about 80,000 molecules, making them one of the most diverse natural products in terms of structure and function. Terpenoids (oxygenated terpenes) and terpenes (hydrocarbons) found in raw, unprocessed foods (Zhou & Pichersky, 2020). Terpenes are categorized by the number of isoprene units (C5H8), derived from dimethylallyl diphosphate and isopentenyl diphosphate as mono (C_10_), di (C_20_), tri (C_30_), sesquiterepenes (C_15_) (Zhou & Pichersky, 2020). Terpenes played a crucial sensory role in foods, contributive to the intrinsic qualities of grape berries and wine (Li, Howell, Fang, & Zhang, 2020), and herbs and spices (Salha et al., 2019).

UFA are highly prone to oxidation, leading to structural alterations that affect their caloric value and bioactivity. UFA are the most abundant and oxidation-prone lipids in dietary fats. PUFA particularly those with easily abstractable protons in the bis-allylic position, oxidize rapidly, even under ambient conditions (Ng et al., 2022). Although lipid oxidation reduces food quality by depleting beneficial FA and producing off-flavors, some oxidation products, like oxylipins, have physiological significance (Knieper et al., 2023). Oxylipins are generated by oxidizing C-18, C-20, and C-22 FA, primarily through enzymes from the lipoxygenase, cyclo-oxygenase, and cytochrome families (Hajeyah et al., 2020). While also present in plants like olives, nitro fatty acids are formed in humans through the electrophilic addition of nitric oxide to UFA in the gastric system when they react with reactive nitrogen species (Sanchez-Calvo et al., 2019). Esterified oxidized fatty acids form TAG or oxidized PL, which may exist free (as oxylipins) or esterified to complex lipids. PUFA-enriched foods are particularly prone to lipid oxidation, especially when exposed to air and heat. Deep-frying significantly alters lipid content and increases oxidized lipids. Storing oats increased their hydroxy and epoxy fatty acids from 0.1 to 2.4 mg/g if initial heating doesn't deactivate enzymes (Yang et al., 2019).

## 3 Dietary lipid nutrition and health studies

Decades of epidemiological, clinical, and experimental research have shown that dietary patterns including Mediterranean diet, and specific food consumption have possessed profound effect on health by preventing from chronic diseases like cardiovascular disease (CVD). Yoshinaga et al. (2021) examined plasma lipidome changes in 12 healthy, young, non-obese women after consuming a high-calorie (1135 kcal) and high-fat (64 g) breakfast. Seventy-three lipid species significantly changed over time, with two distinct subgroups identified at 5 h—slow and fast triglyceride (TG) metabolizers. Slow metabolizers showed elevated TG and phosphatidylinositol (PI) levels, particularly in HDL but not in apoB-containing fractions. These findings suggest that prolonged postprandial lipemia alters HDL lipid composition. Kessler et al. (2020) reported postprandial shifts in TG-linked fatty acid composition, and Averill et al. (2020) found that a high-saturated-fat (HSF) meal enriched HDL in total lipid, triglyceride, and phospholipid content while reducing protein content in 15 adults (60% female, BMI 24.1 ± 2.7 kg/m^2^). Sixteen of 25 phosphatidylcholine species, along with several sphingolipids, increased significantly, whereas apolipoprotein A-I was the only HDL protein to rise. A high-carbohydrate meal caused minimal HDL changes. Hence, HSF meals substantially remodel HDL lipid composition, particularly phosphatidylcholine and sphingolipids, with limited protein alterations. Numerous research have targeted food-derived lipids, including TAG, FA, sterols, and polar lipids, for their biological activity. Each lipid class exhibits multiple bioactivities, with MUFA and PUFA linked to beneficial impacts on organs such as brain, heart, liver and kidney and in the prevention of diseases like CVD, diabetes, and arthritis. Tian et al. (2025) investigated the effect of DHA and arachidonic acid in infant formula on brain and eye lipid composition in a rat model. Results showed that the incorporation of DHA and arachidonic acid has led to significant improvement in the overall cognitive development in infants. Further revealing that supplementation of DHA/ARA between 05-1 enhance mental and psychomotor development index. Sterols also provide health benefits, such as lowering total cholesterol and low-density lipoprotein (LDL) cholesterol, reduced the risk of CVD, and positively influencing immune function and anti-aging (Munawar et al., 2023). Polar lipids, GL, SL, and betaines have shown health-promoting properties, including antioxidant, cardioprotective, anti-inflammatory, and cancer-preventive activities (Jardim et al., 2024). Emerging research underscores the role of FA in various diseases like inflammation, obesity, metabolic syndrome, and cardiovascular disorders. For example, ω-3 PUFAs from flaxseed oil were found to reduce inflammation without affecting the Th2 immune response in allergic conjunctivitis (Hirakata et al., 2019). In terms of obesity, Jiang et al. (2024) evaluated and predicted biomarkers in prediabetic individuals. Weight loss reduced diacylglycerols, ceramides, lysophospholipids, and ether-linked PE, while increasing acylcarnitines, short-chain fatty acids, organic acids, and ether-linked phosphatidylcholine. Specific lipid changes correlated with glycemic improvements, and baseline sphingolipids, diacylglycerols, and triglycerides predicted changes in fasting glucose, HbA1c, insulin, and HOMA-IR. By analyzing plasma LIP and metabolomics, they were able to forecast changes in insulin sensitivity in 104 patients in 8 weeks after intervention of diet-induced weight loss, and baseline lipid profiles. Similarly, Liu, Guo, et al. (2020) observed that male mice consuming a high-fat diet along with 210 mg/kg epigallocatechin gallate for 12 weeks experienced reduced SFA and increased USFA, as well as downregulated hepatic SCD1 and FADS2 expression. It also enriched Verrucomicrobia while reducing Firmicutes and Saccharibacteria, with *Akkermansia muciniphila* showing positive correlation with C18:2 and negative correlation with C22:0. This tea compound also mitigated changes in lipid species, including free FA and TAG, offering protection from negative effect of high-lipid diet. Additionally, a study on the Nordic diet demonstrated its positive impact on the plasma LIP profile of individuals with metabolic syndrome. Gürdeniz et al. (2022) assessed dietary compliance and linked diet-specific metabolic signatures to cardiometabolic risk markers. They indicated healthy Nordic Diet (HND) was characterized by markers of fish, whole grain, and polyunsaturated lipids, while Control diet (CD) reflected lipids with palmitoleic acid. Plasma PC1 scores correlated positively with HDL and inversely with triglycerides while PC2 scores related to higher 2 h glucose, LDL, and triglycerides. However, α-linolenic acid (ω-3) and linoleic acid (ω-6) from soybean and flaxseed oils were found to be less effective in improving metabolic syndrome compared to marine ω-3 PUFA interventions (Jeong et al., 2024). LIP has also contributed to the study of cardio-metabolic diseases, with evidence linking Mediterranean diet interventions to favourable changes in lipid classes and apolipoprotein levels, thus reducing the risk of CVD (Delgado-Lista et al., 2022). An atherogenic diet exacerbates oxidative stress by increasing the production of ROS and impairing antioxidant defenses in peripherical tissues. According to Llanquinao et al. (2024), compared two low-carbohydrate, high-fat diets in SAMP8 mice for 3 months: an atherogenic Cocoa diet (high protein/saturated fat/cholesterol) and the South Beach diet (very high protein/unsaturated fat). The Cocoa diet reduced ROS without affecting energy status or spatial memory, while the South Beach diet increased oxidative damage, altered NMDA receptor composition, and impaired energy and spatial acuity. Furthermore, LIP studies have revealed insights into lipid metabolism in cerebrovascular diseases, such as Alzheimer’s and Parkinson’s disease. Fan et al. (2023) reported that the consumption of higher plasma saturated LCFAs (C14:0, C16:0, C18:0) and no (C20:0) resulted in 1.3–2.2-fold greater risk of progressing from mild cognitive impairment to Alzheimer’s disease. Another research by Nagpal et al. (2019) observed that modified Mediterranean ketogenic diet (MMKD) and the American Heart Association diet (AHAD) produced distinct gut microbiome and SCFA shifts, with MMKD favouring higher propionate and butyrate in mild cognitive impairment. These diet-specific changes influenced amyloid metabolism and cognitive health. Hence, dietary lipids exhibit an important role in preventing and managing various chronic disease.

## 4 Lipidomics analytical techniques

**Fig. 1:**
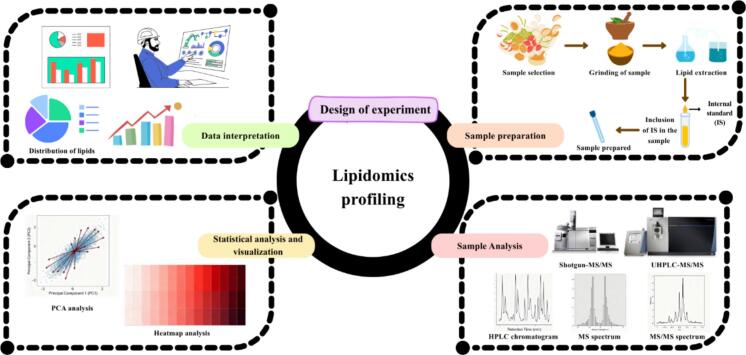
Workflow of lipidomics

LIP identify and quantify more than 1,000 lipid species simultaneously, facilitating comprehensive and robust analyses of lipids with high throughput, sensitivity, and accuracy in different food matrix (Song et al., 2022). The LIP worked on targeted or untargeted compounds based on three steps including pre-treatment (lipid extraction) or sample preparation, data acquisition, and data processing (data analysis) (Fig. 1). While there is some agreement on the different steps in the workflow, getting consistent results across studies is difficult because there aren’t standard methods for analyzing different types of lipids, like neutral, intermediate polarity, or polar ones. The untargeted analysis involve identification and quantification of lipid species over broad range and, consequently, provide specific lipid signature of the food sample. Modern lipid analysis integrates selective extraction, advanced chromatographic or mobility-based separation, and high-resolution mass spectrometry to address three key questions in lipidomics i.e., which lipid classes and species are present, what their fatty-acyl compositions and where structural isomers, particularly double-bond locations occur. Cutting-edge workflows therefore employ orthogonal techniques such as UHPLC, SFC, ion mobility spectrometry (IMS), multi-stage MSⁿ, and specialized chemistries like ozone-induced dissociation (OzID) or Paternò–Büchi (PB) photochemical tagging, all anchored by high-resolution accurate-mass analyzers (Orbitrap, FT-ICR, Q-TOF). Modern sample preparation builds on classical Folch and Bligh & Dyer extractions but increasingly uses MTBE or butanol/methanol systems to minimize class bias, while microfluidic extraction, SPME, and on-tip approaches enable lipidomics from extremely low-input samples or even single cells. The most common extraction methods involve liquid extraction (LE), where the solubility of lipids in organic solvents is influenced by their chemical structures. Hydrocarbon solvents, like hexane, are typically used for neutral lipids without polar groups, while more complex mixtures require solvent systems that combine polar and nonpolar components (Cravotto et al., 2022). Traditional liquid-liquid extraction (LLE) methods, are widely used for lipid extraction in various food samples, achieving thorough extraction suitable for further analysis. Other microextraction methods, including dispersive liquid-liquid microextraction (DLLE) and ionic liquid-based techniques, are effective, particularly in targeted LIP (Pereira et al., 2022). Solid phase extraction (SPE) mostly used alone or in combination with LE to disperse mixtures of lipid into various classes or to concentrate trace lipids. SPE employs cartridges like silica and reversed-phase columns for specific lipid retention. Emerging techniques such as ultrasound-assisted extraction (UAE), solid phase microextraction (SPME), supercritical fluid extraction (SFE), and microwave-assisted extraction (MAE) are also being investigated for extraction of lipid. More environmentally friendly techniques, like methyl tert-butyl ether (MTBE) and butanol/methanol (BUME), are gaining attention, though they have limitations regarding contamination and solvent evaporation. Sometimes, derivatization is applied during sample preparation to improve ionization, volatility, or targeted structural. However, it can be time-consuming and may produce by-products that interfere with analysis. Additionally, accurate quantification requires class-matched isotope-labelled internal standards covering the chemical diversity of the sample matrix. Finally, a pooled quality control sample, composed of equal portions of each sample, is typically included in the analysis to maintain data accuracy and reliability.

Separation science has advanced substantially, For the next step, data acquisition, MS is used priorly for lipid detection due to its high throughput and sensitivity. Recently, high-resolution MS techniques, including quadrupole time-of-flight (Q-TOF), ultrahigh performance liquid chromatography (UPLC)-triple quadrupole-ion trap, and Fourier transform ion cyclotron resonance MS, have become preferred choices for LIP. The most common ionization techniques in MS are ESI, MALDI and atmospheric pressure chemical ionization (Hebra et al., 2020). ESI dominates for polar lipids, APCI is valuable for neutral lipids such as TAGs and sterols, while MALDI and MALDI-2 have revolutionized high-sensitivity lipid imaging at near-cellular resolution; DESI and nanoDESI extend imaging to ambient conditions with minimal sample preparation. UHPLC-based reversed-phase LC (RP-LC) provide high-resolution separation by hydrophobicity and excellent compatibility with ESI-MS for broad untargeted profiling. Hydrophilic-interaction LC (HILIC) complements RP-LC by resolving lipid classes based on headgroup polarity. High-resolution LC-MS is particularly effective for nontargeted LIP, allowing for comprehensive lipid profiling with fast scanning and rich data output, though it may suffer from low stability and repeatability. Recent advancements include single-cell LIP using shotgun MS, which identifies lipid classes and structures with high efficiency (Li, Zhu, et al., 2021). This method, combining chemical derivatization with MS/MS, offers a promising approach for analyzing complex lipid structures in single cells, making it valuable for single-cell biology and precision medicine. Goto-Inoue et al. (2019) used MSI to investigate the localization of TAG molecules in farmed and wild red sea bream. Similarly, Enomoto et al. (2020) employed MALDI-MSI to investigate the distribution of PC species in various porcine tissues, such as transparent tissues, intermuscular fat, and spinalis muscles. Additionally, Enomoto et al. (2020) applied MALDI-MSI to determine the distribution of SM species in pork chops, it was discovered that SM molecules with stearic acid were mostly found in the loin and spinalis muscle, whilst those with palmitic, nervonic, and lignoceric acids were found in transparent tissues. These findings demonstrate how useful MSI is for visualising the distribution of lipid species in foods derived from animals, offering important new information on the spatial arrangement of lipids and how they affect food quality. On the other hand, supercritical fluid chromatography (SFC) has emerged as a fast, environmentally friendly, and highly orthogonal platform particularly suited for nonpolar lipids, isomeric sterols, and complex neutral lipid mixtures. Gas chromatography (GC-MS/FID) remains the gold standard for fatty acid methyl ester (FAME) analysis, providing unsurpassed resolution of fatty acid isomers. GC-MS is used for thermally stable volatile compounds, enhancing efficiency through chemical and electron ionization. However, it requires sample derivatization for non-volatile lipids, which is time-consuming and may introduce errors (Miao et al., 2023). Consequently, GC-FID is employed for targeted analyses of simple fatty acids (Jardim et al., 2024). Shotgun lipidomics—direct infusion without chromatography—offers unmatched throughput but requires HRMS and often ion mobility or MS/MS strategies to overcome isobaric overlap.

High-resolution analyzers are central to modern lipidomics, with Orbitrap, FT-ICR, and Q-TOF instruments providing the mass accuracy and resolving power needed to reduce false positives in complex datasets. Triple quadrupoles remain indispensable for targeted quantification via MRM due to exceptional sensitivity and reproducibility. A major frontier is the integration of ion mobility spectrometry TWIMS, DTIMS, FAIMS, and especially TIMS into lipidomics workflows. TIMS combined with PASEF dramatically increases MS/MS acquisition rates and sensitivity, enabling deep lipidome coverage from scarce biological samples and resolving structural isomers via collision cross-section (CCS) measurements. Structural elucidation has also advanced beyond traditional CID/HCD fragmentation, which cannot localize double bonds. Techniques such as OzID and PB reactions now allow precise determination of C=C locations in intact lipids. OzID leverages gas-phase ozonolysis to produce position-specific fragments, while PB chemistry creates derivatized oxetanes that generate diagnostic ions upon MS/MS. Complementary approaches including UVPD, electron-based dissociation (EID/ECD), and chemical derivatization (epoxidation, DMDS tagging) expand the structural resolution toolkit. These methods can be coupled with IMS or MSⁿ to achieve what is now referred to as “deep lipidotyping.” Imaging mass spectrometry further enriches lipid analysis by mapping spatial distributions of lipid species directly in tissues. MALDI-MSI, boosted by MALDI-2 and improved matrix technologies, provides high spatial resolution for cellular or subcellular lipid mapping, while DESI-MSI enables ambient imaging suitable for certain biological and clinical applications. For quantitative studies, isotope-dilution LC-MS/MS remains the gold standard, particularly in clinical lipid biomarker validation. Untargeted datasets require stringent quality control via pooled QC samples, drift correction, and appropriate internal standards. Data processing now relies on powerful software ecosystems such as MS-DIAL, LipidSearch, LipidHunter, LipidMatch, LipidCreator, and LIPID MAPS databases, many of which integrate MS/MS libraries, retention-time prediction, CCS values, and diagnostic ion interpretation. Specialized tools like LipidOz automate spectral interpretation for OzID and PB workflows, enabling high-confidence annotation of structural isomers. Apart from these tools, to address the needs of diverse and extensive LIP data processing, various new LIP data analysis software packages including MS-Data-Independent Analysis Software (MS-DIAL), XCMS, MetaboAnalyst, MZmine, LipidBlast and LipidIMMS Analyzer, are being used. MS-DIAL handle various data types from DDA and DIA in both GC–MS and LC–MS, and analyze metabolite structures from stable isotope labeling (Takeda et al., 2024). The new version 4.0 complies with the LIP Standard Initiative (LSI) and features the largest lipid database, containing experimental data for 8,051 lipids across 117 subclasses from 1,056 sample runs using 10 types of LC–MS equipment (Tsugawa et al., 2020). XCMS is an opensource R package for metabolomics data analysis which primarily focus on LC–MS data, though it can also handle GC–MS data (Singh et al., 2023). XCMS results can be integrated with other R packages like Mumu package for PCA and multivariable statistics or ggplot2 and heatmap for visualization for advanced data analysis. The XCMS workflow begins with peak extraction and retention time correction across runs, followed by filling in missing peaks and grouping them. Once the data are integrated, the compounds in different samples are quantified based on their peak intensity. MetaboAnalyst process data from GC–MS, LC–MS, and NMR, offered tools for normalization, batch effect removal, functional analysis, and exploratory statistic to generate outputs like volcano plots, heatmaps and KEGG pathways. The recent version, MetaboAnalyst 5.0, features significant improvement and a refactored codebase for enhanced performance (Pang et al., 2024). For LIP, it has introduced a smart name-matching algorithm and expanded its lipid database with 197,854 lipids from RefMet and LIPID MAPS. MZmine 3.0, launched in 2023, enhances metabolite identification using ion mobility data, introduces MS imaging for spatial metabolomics, and supports exporting analysis results in various formats for downstream processing (Schmid et al., 2023). LipidBlast was Lipidomics another open-source database that features 212,516 spectra for 119,200 lipids across 26 classes, including PL and GL, with many complex GL structures published for the first time (Damiani et al., 2023). Most data in LipidBlast have been predicted in silico but validated using 40 types of MS. Lipid IMMS Analyzer offered an online LIP analysis platform that utilized ion mobility for lipid identification, featuring a comprehensive four-dimensional library of over 260,000 lipids (Zhou et al., 2019).

Regulatory frameworks established by authorities such as the European Food Safety Authority (EFSA) and the U.S. Food and Drug Administration (FDA) rely heavily on validated lipid profiling to ensure data quality and support regulatory submissions for food, supplements, and drugs. The importance of validating these analytical methods stems from the need for accurate, reliable, and reproducible results that can be compared across laboratories and studies. For validation, FDA's 2024 guidance on Analytical Procedure Development (Q14) and Validation (Q2(R2)), for instance, provides a general framework for accuracy, precision, to confirm consistent results under repeated testing, specificity, etc. For specific applications, such as novel food approvals by EFSA or LBDDS development reviewed by the FDA, additional validation considerations may apply. For example, the FDA's guidance on liposome drug products highlights specific requirements for qualifying lipid components, while EFSA's nutrient profiling regulations rely on robust data for dietary intake assessments. Overall, the consistent and rigorous validation of lipid profiling methods is a mandatory foundation for building a robust body of evidence that supports regulatory decisions and ensures consumer safety (Guideline, 2022).

## 5 LIP in food application (Quality evaluation in animal fat-meat and fish, plant fat, dairy fat, egg)

*Animal meat*: In animal meat, the major lipid classes include PL, TAG, and DAG, which contain a mixture of SFA, MUFA, and PUFA. LIP has become a critical tool in identifying and quantifying lipids in animal meat, helping to unravel the complex lipid profiles that vary significantly by species, breed, muscle tissue, and a host of environmental factors. LIP analysis enables the evaluation of lipid content in meat, which is affected by parameters such as animal breed, muscle type, diet, breeding environment, and post-slaughter handling and processing methods (Table 3). Man et al. (2021) applied mass spectrometry-based metabolomics to differentiate beef samples from various countries, identifying 24 metabolites as distinguishing markers. According to Nogoy et al. (2022), cattle raised on grass and on grain have dissimilar fatty acid profile. Study have shown that Grass-fed beef contains less total fat and 2,773 mg less SFA per 100 g than grain-fed beef, with lower cholesterol-raising C12:0–C16:0 but also less cholesterol-lowering C18:0. It is richer in trans-vaccenic acid, total n-3 PUFA (EPA, DPA, DHA), and has a lower n-6:n-3 ratio, potentially offering protective effects against cancer and cardiovascular disease. Grain-fed beef, while lower in omega-3 content, has higher total monounsaturated fatty acids beneficial for heart health, and in Hanwoo cattle, grain-fed also contained more EPA and DHA. Slaughter weight significantly influences the lipid profiles in meat. Li, Yang, et al. (2021) identified 592 lipids across 19 lipid classes in finishing pigs, observing that PC and phosphatidylethanolamine (PE) content reduced with higher slaughter weight, and FA arrangements in PC, such as oleic and linoleic acids, were affected by this parameter. Li, Zhu, et al. (2021) investigated the intramuscular fat in longissimus dorsi muscle (LDM), rump muscle (RM) and hamstring muscles (HM) of Dezhou donkey meat using LIP. They indicated with TAG rich in SFA and MUFA being predominant in LDM. Li, Zhu, et al. (2021) compared lipid profiles between LDM and visceral adipose tissue (VAT) in pigs, identifying over 1,100 lipids and finding that LDM possessed higher concentration of GPL and lower levels of glycerolipids. The oxidative stability of beef products may also be evaluated using LIP (Li et al., 2022). Lipid oxidation has been found to occur during the processing stage. Wang, Chen, et al. (2022a) observed 1,541 lipid metabolites in bee pollen, dominated by glycerophospholipids. Drying reduced lipid content and altered TG and FA structures, largely via degradation and oxidation. PCA and OPLS-DA indicated IRD and HAD had the strongest impact on lipid metabolites, while FD had minimal effect. Lipid oxidation was linked to changes in glycerophospholipid, linoleic acid, and glycerolipid metabolism, with PE, PC, PS, PA, and LPC as key lipids. The processing method causes alterations in the FA content of C18:0, C16:1n7, C19:0, and C22:6n3. These substances can lead to rancidity, off odours, and other quality issues in meat products (Huang et al., 2021). Meat products oxidation-associated compounds could be measured and their oxidative stability can be assessed employing LIP (Feng et al., 2022). Similarly, LIP has shed light on the effects of aging meat, which enhances tenderness and alters lipid profiles. Chao et al. (2020) observed that lysophosphatidylcholine (LPC) levels increased by 35% during short-term aging, while extended aging resulted in a tenfold increase in PA. Zhang, Zhang, et al. (2022) demonstrated that fluctuations in lipid content take place throughout the thermal process. High heat processing of chicken flesh results in a decrease in phospholipid content and rise in lysophospholipid levels. The lipid content of meat products, including sausages, may be effectively ascertained using LIP. In processed product like Hengshan goat meat sausages, Jia et al. (2022) quantified 70 key lipids, including Cer, DAG, LPC, PE, PC, PE, phosphatidylserine (PS), phosphatidylinositol (PI), SM, and TAG, using UHPLC-Q-Orbitrap. This approach may be used by researchers to determine the main FA and lipid classes found in various animal products, influence of processing techniques and animal nutrition on the lipid class and FA composition.

**Table 3:** Application of lipidomics in food characterisation

S.No.	Food product	Technique	Lipid type	Key findings	Remarks	Reference
**Animal derived food**						
1	Chicken and pork meat (muscle tissue)	Untargeted 4D lipidomics, incorporating MTBE and MeOH microextraction with UPLC-TIMS-QTOF-MS equipped with ESI	n-3/6 fatty acids, unsaturated triglycerides, and phospholipids	395 unique lipids across four classes and ten subclasses.	PL was found predominantly	Martakos et al. (2024)
2	Egg yolk of hen, duck, goose, quail, pigeon, ostrich and emu	UHPLC-ESI-Triple TOF-MS (Kinetex C18 column) ESI+ and ESI−	PE, LPE, PE-OH, oxPE, PC, PG, PI, PA, LPA, SM	Eight phospholipid classes and 87 molecular species were characterized from yolk of seven egg types	PL profiles of pigeon egg yolks were similar to hen egg yolks, followed by quail, duck, ostrich, emu and goose egg yolks; Based on the PL profiles and the content of DHA + EPA and PUFA hen, goose and pigeon eggs are superior	Zhou et al. (2021)
3	Chicken egg yolk from cage, barn and free-range	LC-MS/MS	LPEs, MAGs, PCs, PEs, SMs, and TAGs	937 lipid species from 20 major lipid subclasses were identified	Lipidomic analysis along with statistical modelling efficiently verifying the provenance of conventional chicken eggs.	Chin et al. (2023)
4	Chicken egg yolk (Deqingyuan, taihe, crossbred, black-boned silky fowl)	UPLC-Q-TOF MS/MS	GP, GL, SP, ST, PK, PR and SL	Identified 1,633 lipid species, 43.78% GP, 25.66% GL, 16.66% FA, 6.86% SP, 4.10% ST, 1.53% 4PK, 1.10% PR, and 0.31% L	Combining chemical analysis with chemometrics offers a convenient and powerful tool to distinguish commercially-available eggs.	Mi *et al*. (2019b)
5	Dezhou donkey meat	LC-MS	TG, PC, PE, GLs, GPs, SLs	Total 1143 lipids where 73 belongs to GLs, GPs, SLs among which 23 were upregulated and 50 were downregulated	Intramuscular fat is responsible for meat flavour, juiciness and tenderness	Li, Zhu, et al. (2021)
6	Cage free chicken egg yolks	High-resolution MS	GPC, GPCp, GPE, GPEp, GPS, GPI, GPG, NAPE, NAPEp, SM, Cer, cPA, MAG, DAG, TAG	Complex structural lipids such as glycerophosphoethanolamines and glycerophosphocholines	Egg yolk is a rich source of structural and energy-rich lipids that possess ω-3 and ω-6 fatty acids an essential precursors of endogenous anti-inflammatory lipid mediators	Wood et al. (2021)
7	Chicken egg yolk	HILIC-LC-MS/MS or UPLC-QTOF MS	GL and GP	A total of 335 individual lipid species covering 23 (sub) classes were identified and quantified.		Wu et al. (2020)
8	Egg	LC-ESIQ-TOF MS	PL, TG	618 lipid species were identified in the yolks of green shell eggs, Tibetan eggs, native eggs, and docosahexaenoic acid eggs	Tibetan and docosahexaenoic acid egg showed higher PL and lower TG	Xie et al. (2020)
9	Pork loins of 1, 8 and 21 days	ESI-MS/MS	Phospholipid, PI, PS	After aging of pork carcass from 1 to 21 days, Total PL quantity decreased 4-folds whereas PI and PS increased by 30 and 73 %, respectively	PI and PS were more resistant to enzymatic hydrolysis compared with the other PL	Chao et al. (2020)
10	Longissimus dorsi muscle (LDM) and VAT from donkeys	non-targeted lipidomics	PC, PE, TGs, PUFA	A total of 1,146 and 1,134 lipids belonging to 18 subclasses were identified in LDM and VAT, respectively, with LDM having higher GP and lower GL contents.	Donkey IMF was rich in GPs and PUFAs distributed preferentially at the sn-1 positions of TGs and sn-2 positions of PC and PE.	Li, Zhu, et al. (2021)
11	Goat meat	untargeted lipidomics approach LC–MS	Cer, DG, LPC, LPE, PC, PE, PI, SM, SPH, PG, PS and TG	12 subclasses of 174 lipids were identified. These lipid variables were involved in the major pathways of GPL and SL metabolism	Significant increases during irradiation were found in total TG, PC, PE, LPE, Cer, LPC and SPH, while the total DG, PS, PG, PI and SM decreased after irradiation.	Jia, Li, et al. (2021)
12	Tan sheep meat	UHPLC-Q-Orbitrap HRMS	sphingomyelin, ceramide, lysophosphatidylcholine, phosphatidylcholine, phosphatidylethanolamines, triacylglycerol	A total of 90 lipids in 6 lipid subclasses were quantified among raw and three types of thermal processed Tan sheep meat	The extent of the losses of SM were greater than the ceramides when boiling	Jia, Wu, Wei, et al. (2021)
13	Longissimus thoracis of finishing pigs	UPLC-Q-Exactive Orbitrap/MS	phosphatidylcholine, specifically oleic and linoleic acids	A total of 592 lipids from 19 lipid classes identified with lipidomics were analyzed.	PC and PE levels decreased with the increase in slaughter weight	Li, Zhu, et al. (2021)
14	Beef	MS-based approach	PCs and PEs	Identified 24 metabolites, 10 metabolites to identify Angus beef samples from others and 7 metabolites to discriminate Australian beef produced by the organic farming	Identify the geographical origin of beef at any point along the supply chain and could be used to develop a verifiable traceability system.	Man et al. (2021)
15	Tan sheep meat	UHPLC-Q-Orbitrap MS/MS	PC, PS, LPS, LPC, PE, PI, LPE, TG, Cer, DG, SM, Sph	Furthermore, 106 significant lipids of 12 lipid classes	The addition of potassium sorbate resulted in higher lipid losses compared with nisin treatment.	Jia, Fan, et al. (2021)
16	Duck meat	Shotgun lipidomics	phospholipid	A total of 118 phospholipid molecules were determined during the water-boiled salted duck (WSD) processing	The effect of spices on most phospholipid molecules began on the first day of dry-ripening, and gradually became more obvious in the subsequent processing. Spice’s main function was to delay the degradation of individual phospholipid molecules.	Li, Zhu, et al. (2021)
17	Black pork (Beijing Heiliu and Laiwu black (BHLB), Duroc×(Landrace × Yorkshire) DLY)	UPLC-Q-Exactive Orbitrap/MS	(PC), triglyceride (TG), sphingomyelin (SM), phosphatidylethanolamine (PE), and ceramide (Cer)	A total of 757 lipids (468 for the positive mode and 289 for the negative mode) were confirmed in pork; 34 and 21 potential lipid markers	monounsaturated fatty acids in BHLB pork were significantly higher than in DLY pork	Li, Zhu, et al. (2021)
18	Hengshan goat meat sausages	UHPLC-Q-Orbitrap	Cer, DG, LPC, PC, PE, PI, PS, SM, TG	9 subclasses of 70 characteristic lipids were quantified.	The decrease of TG concentration was the most significant, from 1072.43 mg kg− 1 in preservative-free samples to 838.53, 786.41 and 681.35 mg kg− 1 in natamycin, potassium sorbate and sodium diacetate treated samples	Jia et al. (2022)
19	Crab (*Portunus trituberculatus*) muscular tissue	HILIC-MS	polyunsaturated phospholipid (PC, PE, PI PS)	38 PMS was semi-quantified including PC, 14.30 mg·g–1, PE, 8.20 mg·g–1, PI, 1.60 mg·g–1, PS, 9.83 mg·g–1	Crab was rich in health-beneficial PL, such as EPA and/or DHA structured PL and plasmalogens, and the muscular tissue	Zhang, Duan, Shang, et al. (2021)
20	Soft-shelled turtles	HILIC-MS	phospholipids	Total of 55 phospholipid molecular species were identified	This method also can be used for authenticating different strains of soft-shelled turtles	Yu et al. (2019)
21	Salmon muscle tissue	ESI-MS/MS spectrometry	TAG	A total of 98 TAGs were identified.	The predominant TAG species were 16:0–18:0–20:5 (10.4%), 18:1–18:2–22:6 (9.0%), and 18:0–18:1–22:6 (16.4%) in salmon muscle tissue	Yeo and Parrish (2020)
22	Seaweeds	LC-MS	GL, PL, and betaine lipids	In total, 477 different species of polar lipids were identified and distributed over the six seaweeds.	Overall, the present results allow better understand the specificity of the polar lipidome presented by different species of seaweeds and to contribute to their valorization framed by a blue bioeconomy.	Lopes et al. (2021)
23	Marine aquaponic (*Salicornia ramosissima* and *Halimione portulacoides*)	LC-MS and MS/ MS	Phospholipids, glycolipids	Phospholipids and glycolipids were identified and quantified	Halophytes produced in aquaponics have higher levels of glycolipids with n-3 fatty acids. In the case of H. portulacoides, a significant increase of phospholipids bearing n-3 fatty acids (most in PC and PE) was also recorded.	Maciel et al. (2020)
24	Clam (*Corbicula fluminea*)	graphene/ fibrous silica nanohybrids based solid-phase extraction and HILIC-MS analysis	phospholipids., PE, PC	total of 35 PMS in clam were identified and quantified, and 53.62% EPA/DHA structured PC molecular species were obtained.	The ions of PE 16:0/18:1 (m/z 716.4), PC 16:0/20:5 (m/z 824.6) and etc. were regarded as the main ion responsible for the difference between the three samples	Song et al. (2021)
25	Shellfishes (mussel, abalone, scallop, oyster, clam and razor clam)	hydrophilic interaction chromatography mass spectrometry	phosphatidylcholine plasmalogen (plasPC), phosphatidylethanolamine plasmalogen (plasPE	A total of 19 plasmalogen molecular species were successfully identified, including nine phosphatidylcholine plasmalogen (plasPC), seven phosphatidylethanolamine plasmalogen (plasPE), and three phosphatidylserine plasmalogen (plasPS).	The quantitative results indicated that mussel (32 μg·mg−1) possessed the highest content of plasmalogens, followed by oyster (21 μg·mg−1) and razor clam (15 μg·mg−1).	Wang et al. (2021)
26	Tilapia (*Oreochromis niloticus*) (muscle, head and viscera)	UPLC-ESI-Q-TOF-MS	FA	622, 530 and 513 lipids were identified; Five FA and 33 lipid species were considered as the potential biomarkers.	TAG were the predominant fraction; PUFA had higher percentages in PL (30.35–52.05% of total FA) than in TAG (18.11–25.15%).	He, Wu, et al. (2021)
27	Salmon and Rainbow Trout	Rapid Evaporative Ionization Mass Spectrometry-Based Lipidomics	PE and PC	A total of 12 fatty acids and 37 phospholipid species was identified	Ions with high correlation values, such as of m/z 747.50, 771.49, and 863.55, indicated large weights in identification of the salmon and rainbow trout.	Song et al. (2019)
28	Marine	Ultraperformance Liquid Chromatography Coupled with Quadrupole Time-of-Flight Mass Spectrometry	Glycerolipids, PLs	700 molecular species from 12 major lipid subclasses were identified.	Glycerolipids (73.7−85.6%) and phospholipids (PLs, 13.7−25.6%) were dominant components in total lipids. Polyunsaturated fatty acid PLs, such as phosphatidylethanolamine (PE, 16:0−22:6), PE (18:1−22:6), and phosphatidylcholine (16:0−22:6), were the major molecular species in PLs.	Wang, Yang, et al. (2019)
**Plant derived food**						
29	Milk (donkey, cow and human milk)	UHPLC-Q-Exactive Orbitrap Mass Spectrometry	phospholipid (PL), TG	87 significantly different lipids (SDLs) were found between DM and HM, and 77 SDLs between DM and CM and these SDLs were involved in 21 metabolic pathways.	HM had a higher proportion polyunsaturated TGs and LCFAs compared with CM and DM. The percentage of saturated TGs in CM was higher than that of DM and HM. For polar lipids, the content of PLs in DM was much lower than that of CM and HM a	Zhang, Li, Duan, et al. (2021)
30	Human, bovine and caprine milk	UHPLC-Q-TOF-MS and GC-MS	TG, DG, SM, PC, 10 Cer, HexCer, Hex2Cer, PE, PG, PS, PI, PA and CL	A total of 13 lipid classes were analyzed; A total of 215 and 147 lipids were identified as potential biomarkers	Human milk was 11 richer in TG containing LA, SM containing ULCFA and PLs containing ARA, DHA 12 and DGLA	Wang, Yang, et al. (2019)
31	Bovine Colostrum and Mature Milk	UHPLC-QTOF-MS Lipidomics	PEs, PG, TGs, CL, DGs, Hex2Cers, HexCers, PAs, PC	335 lipids assigned to 13 subclasses were characterized and 63 significantly differential lipids (SDLs) were identified.	Among the 63 SDLs, the levels of 21 lipids were significantly lower in BM than in BC, including 5 PEs, 1 PG, and 15 TGs. The levels of the remaining 42 lipids increased in BM, including 1 CL, 9 DGs, 9 Hex2Cers, 3 HexCers, 3 PAs, 2 PCs, 12 PEs, and 3 TGs	Li et al., (2020)
32	Milk (human, horse, goat and cow)	LC/MS-based		37 significantly different metabolites (P1) were identified in the four milk samples; revealed seven main metabolic pathways were identified.	The content of inositol, 2-Oxobutanoate, d-Glucosamine, d-Glucose, and xylobiose in human milk samples were significantly higher than those of the other three milks, while the content of l-Valine, creatine, betaine, hippuric acid in human milk were significantly lower than those of the other three milks.	Wu, Wei, et al. (2021)
33	Human milk (HM) and formula milk (FM)	LC–Q-TOF–MS	LC-PUFAs, sphingomyelines, glycerophosphoethanoloamines	Long-chain (PUFAs) containing lipid species of were detected in HM collected in all studied lactation stages as compared to FM.	FMs contained a higher content of MCTGs and SCTGs than HM; TG not present in HM; PL containing LC-PUFAs are present in the HM of all lactation stages.	Hewelt-Belka et al. (2020)
34	Olive oil after hydrolytic reaction	RP-C18 HPLC coupled to negative polarity HRMS	PLs and FFAs	A total of 24 polar lipids, comprising 19 phospholipids and 5 sulfolipids, and 27 free fatty acids were tentatively identified. After one month storage of oil, more than forty compounds were identified, due to hydrolysis and oxidation reactions.	Among the hydrolysis reactions, phosphoester hydrolyses seem to be faster than glycerol ester ones.	Capriotti et al. (2021)
35	Beans (chickpea and soybean)	UHPLC-Q-HRMS	Phosphatidylcholine, PUFA, DHA	A total of 49 molecular species were identified by UHPLC-Q-HRMS.	PC content of chickpea (*Cicer arietinum*) and soybean (*Glycine max*) was 50.0 and 34.0 mg/g, respectively; soybean contained high proportion of PUFA (58.78%), and chickpea contained high proportion of docosahexaenoic acid (DHA) (2.73%)	Yang et al. (2020)
36	Mushrooms	UHPLC-QE Orbitrap/MS/MS	PE, LPE, PS, and LDGTS	20 lipid classes and 173 molecular species were identified and quantified.	LPE and Cer non-hydroxy fatty acid dihydrosphingosine as a potential biomarker.	Zhou et al. (2021)
37	Green, Yellow, and Red Bell Peppers	liquid chromatography mass spectrometry	β-cryptoxanthin	8000 lipid compounds were detected with 315 compounds (106 annotated) found in all three colours.	The compound most strongly associated with colour was the carotenoid, β-cryptoxanthin	Sutliff et al. (2021)
38	Lupin Seeds	Liquid Chromatography and Tandem Mass Spectrometry	Phospholipids, Lysophospholipids	200 main phospholipids were regiochemically identified; including 52 PC, 42 PE, 42 PA, 35 PG, 16 LPC, 13 LPE, and 10 PI, is reported.	Whereas 18:1 and 18:2 acyl chains were present in the most abundant molecular species of PI, PG, PE, PC, and LPC, polyunsaturated acyl chains 18:2, 18:3, 19:2, and 19:3 were the most abundant in PA.	Calvano et al. (2020)
39	Flaxseed oil on roasting	UPLC-Q-Exactive Orbitrap mass spectrometry	Phospholipid, triacylglycerol, PC, PE, PG, PL etc	238 lipids including fatty acid (45 species), phospholipid (37 species), triacylglycerol (125 species), and oxidized fatty acid (21 species) were determined in unroasted and roasted flaxseed oils. 23 lipids were determined as potential biomarkers	PE, PC, phosphatidylglycerols, PI, LPC, LPE, and oxidized FA firstly increased and then decreased during roasting	Zhang, Li, Yang, et al. (2021)
40	Banana (*Musa cavendish*) peel and pulp at unripe, ripe and overripe stage	LC-MS/MS	PCs, LPCs, PEs, PE-(O), PE-(P)s, LPEs, PIs, SMs, PSs, PGs, BMPs, Cer, COH	366 lipid species, a total of 143 lipid molecules were detected	Major lipid class identified in pulp	Sun, Wang, et al. (2020)
41	Rice (fresh and stored)	UPLC-Q-extractive orbitrap mass spectrometry	DG, TG, LPC, PC, PE, PG, PI, cardiolipin, Cer, hexosylceramide, dihexosylceramide, trihexosylceramid, sitosterol ester, acyl hexosyl campesterol ester, acyl hexosyl sitosterol ester, digalactosyldiacylglycerol, monogalactosyldiacylglycerol, monogalactosylmonoacylglycerol, and sulfoquinovosyldiacylglycerol	A total of 21 subclasses of 277 lipids including fatty acid (36 species), (O-acyl)-1-hydroxy fatty acid (6 species) etc, were first identified in rice during storage	FA and OAHFA increased, whereas PC, PE, and PG decreased in both rice varieties during storage	Zhang, Duan, Shang, et al. (2021)
42	Raw and roasted macadamia nuts	Shotgun-NL-ESI-MS/MS	FA (Myristic acid, Palmitic acid, Palmitoleic acid, Tetracosanoic acid etc), TAGs	Identified 38 lipid molecular species in macadamia nuts, which were characterized and quantified, including 28 TAGs and 10 FFAs	Significant increase in FFA content during roasting both raw and roasted macadamia nuts have high nutritional value	Tu et al. (2021)
43	Irish Ale	LC-MS	PC, ALA, EPA, MUFA	Several bioactive diacyl and alkyl-acyl PC molecules containing n-3 PUFA, mostly DHA followed by ALA and EPA, and MUFA such as OA were identified	Several SL and GL with a wide range of bioactivities and health benefits were also identified	Tsoupras et al. (2020)
44	Green tea	UHPLC-Q-Exactive/MS	PA, PC, PE, PI, PG, PS, LPC, MGDG, DGDG, SQDG, DG, TG, Cer, GlcCer,	283 lipid species were detected, covering 20 subclasses.	Decrease of PAs content during green tea manufacture was identified for the first time. Significant lipidomic variations were observed during green tea manufacture	Li, Zhu, et al. (2021)
45	Green Arabica coffee beans extracted by matyash method, folch method and bligh and dye method	LC-HRMS/MS	PC, LPC, PE, and LPE	Matyash (MA) method, (131 lipids) compared to the other methods	Immature beans contain lower levels of C-5HT, PI, PC, LPC, PE, and LPE than mature and over-riped beans The MA method yielded the greatest number of lipid compounds and considered the most suitable for the lipid extraction of green coffee bean	Silva et al. (2020)

*Marine meat*: Fish lipids, a primary source of essential FA, have garnered increasing attention due to their health benefits. They provide significant amounts of n-6 FA, particularly arachidonic acid, and n-3 FA, such as EPA and DHA, which are vital for infant development, brain function, and inhibition of hyperlipidemia (Bettadahalli et al., 2020). The composition of lipid varies significantly depending upon the species. Recent LIP studies have deepened the understanding of lipid profiles in fish and shellfish (Table 3). In order to characterise bioactive lipids for species separation and nutritional evaluation, Wang, Zhang, et al. (2019) identified 12 lipid subclasses in 1 freshwater and 3 marine fish species, which included over 700 lipid species. Even though UPLC-ESI-Q-TOF-MS is a very thorough approach, it does not reveal the position of double bonds in fatty acids. Using UPLC Q-TOF-MS/MS, Wang, Yu, et al. (2024) found 449 lipid molecular species from 13 subclasses in marine fish roe. They emphasised that mackerel roe and yellow croaker have a significant nutritional benefit due to their high n-3 PUFA-PL content. In a thorough LIP analysis of tilapia, He, Cao, et al. (2021) looked at the fish's head, viscera, and muscle. They discovered that the head and viscera had more lipids than the muscle, and that TAG made up the majority of the total lipids, accounting for more than 80% of the total lipids. These findings suggest the potential application of tilapia lipids, particularly C52:2 and C52:3 TAG, in infant food. Wang et al. (2021) identified plasmalogen lipids in six edible shellfish using HILIC-Q-TRAP-MS, emphasizing their relevance to brain and heart health. Song et al. (2021) synthesized a method for solid-phase extraction of PL in clams, and identified 35 PL molecular species and noting the abundance of EPA/DHA structured PC molecules.

*Egg*: Egg yolks are rich in essential lipids that play crucial roles in membrane structure and cell signaling. Recent advancements in LIP have enabled a comprehensive characterization of lipid profiles in eggs, focusing on component analysis, breed and sex differentiation, and the effects of feed. The fatty acid content of eggs from caged chickens differs from that of eggs from free-range hens. One important macronutrient is high in calories is fatty acids, namely n-3 PUFA. Zhou et al. (2021) identified and quantified eight PL classes and 87 molecular species from 7 different types of eggs including pigeon, emu, hen, quail, goose, duck, and ostrich using UHPLC-ESI-Triple TOF-MS. Their findings exhibit variation in the molecular species and concentrations of PL between pigeon and hen egg yolks compared to other types of egg yolks. Chin et al. (2023) differentiated lipid profiles among cage, barn, and free-range eggs using LC-MS/MS, identifying 937 lipid species across 20 major subclasses, predominantly comprising acylglycerides, PC, and PE. Tomaszewska et al. (2021) noted that the lipid composition of egg yolks would vary depending on how the chickens are grown, whereas Luo et al. (2023) found that the constituent of TAG, PL, and sphingolipids in egg yolk dropped considerably with increase in storage time. Fatty acids that were esterified to the glycerol backbone of PL ranged from C16:0 to C22:6. On the other hand, fatty acids esterified to TAG ranged from C14:0 to C20:0. After the former displayed higher levels of PC (O-34:0), PC (34:1), and PE (34:1), there were notable differences in the PL profile between eggs from chickens kept on the free range and fed vegetable-based diet and eggs from the other conditions. Mi, Shang, Zhang, and Fan (2019) detected 1,633 lipid species in egg yolk, revealing distinct lipid abundance and FA side chain compositions among crossbred black-boned, Deqingyuan and Taihe eggs. Xie et al. (2020) utilized UAE combined with isopropanol to identify 618 lipid species in yolks from various egg types, noting that Tibetan and DHA eggs had higher PL and lower TAG concentrations. Their analysis suggested that hen eggs might be the best choice regarding taste and price. Additionally, Wood et al. (2021) highlighted the presence of glycerophosphoethanolamines and glycerophosphocholines in egg yolks, reinforcing their status as a rich source of complex structural lipids vital for lipid homeostasis. Furthermore, dietary supplementation with various n-3 PUFA can significantly alter the FA composition of egg yolk, enhancing its nutritional value. Wu et al. (2020) demonstrated that supplementing hens with flaxseeds and other n-3 PUFA sources led to a rise in TAG levels and essential fatty acids. They identified a total of 335 individual lipid species across 23 subclasses, finding that dietary α-linolenic acid (ALA) primarily accumulated in the TAG fraction, while synthesized or preformed DHA was mainly found in GPL. LIP has been exploited by researchers to recognize the distinct lipid profile of salted duck eggs and evaluated how salting techniques influences the lipid composition. According to LIP study, eggs of salted duck are a good source of lipids, such as cholesterol and phospholipids, and the salting procedure cause changes in the composition and functional characteristics of egg lipid. Additionally, 315 lipids, including glycerolipids, GPL, glycosphingolipids, and neutral glycosphingolipids, were found in egg samples after Harlina et al. (2021) studied LIP profiling in eggs treated with clove extract.

*Dairy*: Milk metabolites vary significantly across dairy species, such as cows, goats, and horses, influencing the nutritional value of infant formulas. Bovine milk lipids play essential biological roles, impacting human health and food functionality. Variables such as nutrition and genetical buildup of the dairy animal along with the processing techniques, affect the FA content of dairy products. For instance, FA composition of dairy products derived from animals fed grass differs from that of dairy products derived from animals fed grain. The oxidative stability of dairy products may also be judged by employing LIP. Li et al. (2019) utilized LIP to study difference in lipid composition between bovine colostrum and mature milk, and identified 335 lipids and 7 metabolic pathways associated with variations in lactation, with GPL metabolism being the most prominent, followed by the SL metabolism and glycerolipid metabolism. Wu, Chen, et al. (2021) characterized milk metabolites from human, cow, horse, and goat milk and revealed 37 significantly different metabolites. Understanding these variations can enhance the evaluation of milk properties and improve the formulation of infant formulas that closely resemble human milk. Similarly, Wang et al. (2020) investigated the lipid and FA compositions of human milk (HM), bovine, and caprine milk, identifying 13 lipid classes and potential biomarkers, aiding in infant formula design. HM acted as an optimal source of nutrients for newborns, and formula milks (FMs) are designed to replicate its composition by incorporating various lipid sources. Hewelt-Belka et al. (2020) performed comprehensive comparison of HM lipid compositions across lactation stages and various age-targeted FMs, revealing significant qualitative and quantitative differences in lipid classes. Furthermore, donkey milk (DM) is proposed as a hypoallergenic alternative to cow milk (CM) for infants with protein allergies. Zhang, Duan, Shang, et al. (2021) compared the lipid profiles of DM, HM, and CM using UHPLC-MS/MS, noting distinct differences in PL and TAG. This study highlights the potential of DM in developing infant formula. Additionally, Deng et al. (2022) examined lipid variations in mare's milk based on different feed types, finding higher levels of specific lipids linked to fatty acid metabolism. Xia et al. (2020) used UPLC-Q-TOF-MS to analyse lipid subclasses in mare's milk, reported variation in raw and fermented forms.

Lipid in cheese composed of saturated and unsaturated FA, waxes, and phospholipids. These substances can cause rancidity, off odours, and other quality issues. LIP has made it possible to identify the unique lipid profiles of different cheese varieties, including blue, mozzarella, and cheddar, along with variables that affect the composition of cheese lipid, including the kind of milk use, the addition of starter cultures, and ageing techniques. The fat content of cheese has been associated to it’s flavour, texture, and volatility in addition to possible health advantages such its anti-inflammatory and anti-tumour. LIP can detect fat alterations that occur throughout the processing of dairy products; these changes serve as a benchmark for quality control of the final product. Jia et al. (2021) clarified that the fat content of fermented goat milk changes following fermentation, samples of brown goat milk showed a considerable rise in organic acid, peptide, and MCFA and LCFA concentrations. A total of 174 lipids and 108 metabolites linked with sensory quality were found. As Maillard reaction intermediates, heterocyclic chemicals altered the colour, taste, and odour of fermented brown goat milk, whereas variations in the triglyceride content dropped the effect of the off-odour, significantly enhancing the sensory qualities. Zhang, Zhang, et al. (2022) describe how fat content of milk is changed when subjected to hight thermal temperatures during course of processing. Additional lipid oxidation reactions and a decrease in the quantity of mild oxidation products were the outcomes of heat treatment. Furthermore, UHT-treated milk may be identified by its quantities of free FA, especially oxidised free FA, and lysophospholipids. In turn, raw, pasteurised, and ESL milk may be distinguished using oxidised phosphatidylcholine, oxidised PE, ether-linked PE, diacylglycerol, triacylglycerol, and oxidised triacylglycerol.

*Plants*: In plant, lipids are classified as FA, glycerolipids, GPL, SL, sterol lipids, prenol lipids, saccharolipids, and polyketide, covering nearly 43,413 lipid molecular species. Recent studies using advanced MS technique including ESI-MS/MS, MALDI-TOF-MS, Orbitrap MS, and MS imaging have provided detailed lipid profiles across different plant-based foods. Capriotti et al. (2021) identified long-chain free fatty acids (FFAs) at trace levels in extra-virgin olive oil (EVOO) using shotgun LIP, finding significant lipid degradation after one month of storage due to hydrolysis. Alves et al. (2019) analysed TAG and polar lipid profiles of olive pulp using C_30_ reversed-phase LC and normal-phase hydrophilic interaction LC, respectively coupled with ESI-MS and ESI-MS/MS, identifying 71 TAG ions and over 350 molecular species, including 107 polar lipids across 11 classes comprised of PL, glyceroglycolipids, glycosphingolipids, and betaine lipids. Similarly, Martin et al. (2023) used LIP techniques to analyse lipid profiles of lipid biosynthesis in oil palm during fruit development. Yang et al. (2022) observed the variation in the seed oils of flaxseed, *Hibiscus manihot L.*, and sunflower using LIP, while Sun et al. (2022) monitored lipid dynamics during storage of hazelnut oil. They identified 103 lipids in hazelnut oil over a 24-day storage period, with significant decreases in TAG, DAG, PA, PE, phosphatidylethanol, Cer, and total lipids. Cui et al. (2024) examined the lipid profiles of 5 mango kernels using LIP and chemometrics. They identified 900 lipids, with 9cC18:1 and C18:0 as the major FA across all samples. UFA were higher in Hongyu mango (HYM), Australian mango (AM), Tai mango (TM) and Jidan mango (JDM), while Qingpi mango (QM) had more saturated FA. AM and JDM showed the maximum GPL and saccharolipid contents. Wang, Zhong, et al. (2022) used UHPLC-Q-Exactive Orbitrap/MS for measuring 7 walnut varieties from Xinjiang, identifying 390 lipids across 6 categories and 30 subcategories. Glycerolipids and GPL were abundant. Dried walnuts (DW) had higher lipid content than fresh walnuts (FW), with reduction in some subcategories like MGDG and PC and enhancement in LPC and MePC. A total of 128 lipids as potential markers were identified to differentiate between FW from DW. Huang et al. (2022) analysed the LIP of hickory (*Carya cathayensis*) nuts in their embryogenesis using UPLC-MS/MS, and detected 544 lipid species. Hou et al. (2022) used desorption ESI with ion mobility and Q-TOF MS to map lipid distributions in eight edible nuts. Negative ion mode primarily detected glycerophospholipids, while positive mode identified glycerolipids and phosphatidylcholines. A total of 87 compounds including 47 glycerophospholipids, 24 glycerolipids, alkyl phenolic acids, fatty acid acyl metabolites, oligosaccharides, and amygdalin were characterized, with collision cross-sectional values measured. The nut cotyledon’s outer shell contained more abundant components, whereas hydrolyzed glycerophospholipids were richer in the center. These spatial insights enhance understanding of nut metabolite localization. Li, Hua, et al. (2021) employed nontargeted LIP using UHPLC-Q-Exactive/MS to characterize lipid changes throughout green tea manufacture. They identified 283 lipid species and notable variations associated with degradation of chlorophyll, GL and decrease in PL, all of which contribute to the colour and aroma quality of tea. Similarly, lipid composition in coffee bean also plays crucial role in maintaining brew quality. The lipid composition primarily consists of TAG with smaller amounts of PL and βN-alkanoyl-5-hydroxytryptamides (C-5HT). Silva et al. (2020) evaluated three extraction methods—Bligh-Dyer (BD), Folch (FO), and Matyash (MA)—and analyzed the lipids using LC-HRMS/MS. They identified 131 lipids using the MA method, providing valuable insights into lipid composition of coffee and its relationship with quality.

LIP has also been employed to explore lipid metabolism in rice. Concepcion et al. (2020) utilized untargeted LIP to investigate the genotypic effects of lipids on cooking and eating quality in a rice mapping population. Thousands of rice grain lipids were detected and categorized into six groups: fatty acyls, glycerolipids, GPL, SL, sterol, and prenol. Lipid profile differences between waxy and non-waxy rice were evident, with strong correlations found between specific lipids, amylose content, and viscosity, particularly those forming the amylose-lipid complex. Complexing of amylose with all the fatty acids in rice flour increased with the increase in cooking time. Myristic acid had the highest ability to form the complex with amylose and stearic acid the least (Kaur & Singh, 2000). Amaranth grains were reported to be richer in proteins and lipids than cereals, but their unsaturated fatty acids were prone to oxidation. Removal of lipids improved shelf life and functional properties of amaranth flours. Removal of lipids from flours increased final viscosity and stability (lower breakdown viscosity) indicated defatting a value-added process (Shevkani et al., 2014). Zhang et al. (2021) used LIP (UPLC-Q-Exactive Orbitrap/MS method) to examine the lipid changes in rice during storage for 360 and 540 days, detecting significant difference among 22 lipids on storage. Beans belong to the Leguminosae family (*Phaseoleae*, subfamily *Papilionoideae*) and are rich in various nutrients, including vitamins, minerals, complex carbohydrates, protein, dietary fiber, and a relatively low content of lipids. Despite their low lipid content, epidemiological data suggest that the high quality of bean lipids confers health benefits. Yang et al. (2020) identified PC molecular species in 6 types of beans using UHPLC-Q-HRMS, revealing PC as abundant in adzuki bean, soybean, common bean, and runner bean, while chickpea was noted for its potential as a dietary source of DHA and ether lipids, highlighting the need for further research to improve the nutritional value of beans and their products. Calvano et al. (2020) characterized phospholipidome in yellow lupin (*Lupinus luteus*) seeds using HILIC and ESI and identified PC as the most abundant (41 %), followed by LPC, PE, phosphatidylglycerols (PG), PA, PI, and lysophosphatidylethanolamine (LPE).

Furthermore, few studies were also undertaken on various fruits and vegetables, Yang et al. (2021) extracted total lipids from eight wild edible mushrooms using UHPLC-Quadrupole-Exactive Orbitrap MS and identified LPE (16:1) and ceramide non-hydroxy FA dihydrosphingosine (d23:0−10:0) as biomarkers, aiding in the nutritional assessment of wild edible mushrooms. Bell peppers (*Capsicum annuum*), known for their antioxidant properties, were analysed by Sutliff et al. (2021) for LIP differences in colour-associated compounds among 23 samples of green, yellow, and red varieties. They identified 8,000 lipid compounds which further enhance the understanding of how these compounds influence health outcomes.

## 6 Impact of food processing on lipid profile evaluated by different LIP approaches

Food processing operations significantly impact the lipid profile of various food matrices. Lipids undergo alteration in structure and composition at different processing conditions. The primary lipid classes affected by processing include PL, TAG, and PUFA. LIP plays an important role in identifying these changes and their implications on food quality and safety. In studies of meat and seafood, chemometric tools like PCA and OPLS-DA have been used to differentiate between lipid profiles of raw and processed samples. During cooking like frying, air frying, roasting, boiling, baking, etc., lipids undergo oxidation, hydrolytic and polymerization reaction, etc which lead to formation of various desirable and undesirable compounds like aldehydes and ketones. Hu et al. (2023) used LIP to assess the volatile profiles of palm, soybean, rapeseed, and flaxseed oils under thermal conditions (temperature 100, 150 and 200°C). The study highlighted potential markers such as palmitic acid (undecanal, dodecanal and 2-hexanone), oleic acid (2-undecenal), and linoleic acid ((*E*,*E*)-2,4-nonadienal, 3-octen-2-one, and 3-nonen-2-one), which provided insights into the oils' stability during processing. This understanding allows the selection of oils based on their suitability for high or low-temperature cooking. Liu et al. (2022) analyzed aldehydes in 10 commercial oils during frying at 180 °C using headspace-GC/MS and observed increased level of aldehyde over time with four key aldehyde recorded were pentanal, hexanal, (E)-hept-2-enal, and nonanal. Additionally, shotgun ESI-tandem MS enabled efficient profiling and quantification of cold-pressed rapeseed oils, specifically after microwave processing, by developing methods to analyse TAGs, PLs, and FFAs (Xie et al., 2019). They indicated non-significant changes in FA and TAGs while significant increase by 40 fold in FFAs and PLs. Xiao et al. (2022) employed LIP to identify 706 lipid metabolites in high-oleic acid peanut seeds, showing significant variations in lipid species across the three processing techniques (boiling, baking and frying). Among boiling, baking and frying groups, 75, 175 and 242 lipid metabolites were differentially expressed, respectively. Boiling was found to retain higher levels of beneficial lipids and antioxidant activity, whereas frying led to an increase in PUFA like FA/C18:2, emphasizing the role of LIP in evaluating the nutritional impact of food processing. Similarly, Jia, Wu, Wei, et al. (2021) studied the effects of boiling, steaming, and roasting on Tan sheep meat using UPLC-QOrbitrap HRMS. They quantified 90 lipids in six subclasses and concluded that boiling was the most suitable method for atherosclerosis patients due to a greater loss of SM than Cer. Steaming, on the other hand, was found to be more suitable for the elderly and infants, as it resulted in smaller losses of PC and LPC. Air frying (AF) has appeared as a healthier choice to deep-oil frying, reduces fat content while maintaining sensory properties like crispiness. Zhou et al. (2022) observed 4.26–6.58 g/100 g of fat content in AF shrimp which was lower when compared with deep fried shrimps samples. Redfern et al. (2021) highlighted the complex reactions, particularly lipid oxidation, that occur in lipid-rich foods like salmon during cooking. In a LIP study, Song et al. (2024) identified 773 differential expression metabolites (DEMs) in air-fried salmon, including GPL, glycerides, and SL as key metabolites. Of these, 34 DEMs with p-values < 0.05 were linked to linoleic acid, glyceride, and GPL metabolism pathways. Correlation network analysis highlighted that specific DEMs, such as PC, LPC, TAG, FA, and PE, were strongly associated with lipid oxidation. As temperature increased, significant reductions in PC and TAG with UFA were observed. Furthermore, cooking processes significantly alter the lipid composition of meats, affecting their flavour profile and stability. Li et al. (2022) described how lipids serve as precursors to volatile aroma compounds during cooking, with PL and TAG playing critical roles. Li et al. (2022) conducted an in-depth analysis of the lipid composition changes in donkey meat during cooking, identifying 992 lipids in raw donkey meat (RDM) and 1,022 in cooked donkey meat (CDM), belonging to 12 lipid subclasses. The study revealed that 116 lipids exhibited significant differences between RDM and CDM. Notably, 61 TAG rich in SFA and MUFA were retained in CDM, while 37 GPL rich in PUFA decreased in abundance. This suggests that TAG is key to retaining SFA and MUFA during cooking, which may contribute to aroma retention, while GPs, associated with PUFA, may play a crucial role in aroma generation.

Roasting is commonly used thermal cooking method used to enhance bioactive components, flavour, and oil quality. However, the quality varied depending upon the degree of roasting (applied temperature and time). The light roasting minimally affects colour and flavour, while dark roasting yields darker oil with a stronger flavour. Peanut oils subjected to roasting show increased antioxidant activity and oxidative stability, although fatty acid composition remains largely unchanged (Zhang et al., 2020). Suri et al. (2019) found that peanut oil yields increased significantly with both mechanical (41.18 to 46.28 %) and solvent extraction (47.77 to 55.35 %) after dry air roasting. They indicated that the roasting process disrupts cell membranes in peanuts, leading to the rise of PL and escape of intracellular TAG. Zhang, Guo, et al. (2022) used UPLC-Q-Exactive Orbitrap mass spectrometry to study the lipid profile changes in peanut oil during light and dark roasting at 180 °C for 10 and 40 min, respectively. The study revealed that light roasting released intracellular TAGs and PL, while dark roasting led to increase in PL as well as degradation of PE and oxidized fatty acids, which contributed to the characteristic flavour of roasted oil. In flaxseed oil, roasting increases PL content initially, but prolonged exposure to high temperatures degrades these lipids, resulting in the loss of co-products that could be otherwise valuable (Zhang et al., 2021). Liu, Gao, et al. (2024) mapped lipid profiles in roasted quail meat using LIP, identifying PL, neutral lipids, and SL as key markers. Lipids with unsaturated C18 acyl groups were crucial for aroma formation, with neutral lipids playing a major role in aroma retention. These findings suggest that roasting significantly alters lipid composition and contributes to flavour development in various food products. Shi et al. (2019) conducted an untargeted metabolomics analysis using the UPLC-Q-Exactive Orbitrap MS approach to screen lipid species in tilapia fillets during different cooking methods—steaming, boiling, and roasting. The study identified eight key lipid species variables (LPS, LPG, LPI, DG, LPC, TAG, LPE, and Cer) and 137 individual lipids that exhibited substantial variation among different cooking method. Among the cooking methods, steamed and boiled tilapia fillets were found to offer higher nutritional value in terms of the recommended daily intake of EPA and DHA, making them preferable options compared to roasted fillets. Roasting hazelnuts induces changes in polar lipids, including PL and GL. Cerulli et al. (2023) used LC-ESI/HRMS, LC-MS/MS, and NMR to characterize polar fraction in raw and roasted hazelnut. Roasting resulted in threefold higher polar lipid including phospholipids, glycolipids, sphingolipids, oxylipins. Additionally, Lyso-phospholipids and phospholipids dominated both fractions (94–97%).

Besides cooking, other processing operations such as drying, curing, fermentation also affect lipid classes, influencing not only the nutritional value of food but also their sensory characteristics. Drying bee pollen, an essential preservation method, results in lipid oxidation and degradation. Song et al. (2020) stated an increase in lipid oxidation when dried at higher temperature for longer duration. Wang, Chen, et al. (2022a) used LIP to compare the lipid profiles of bee pollen during various drying techniques like infrared drying (IRD), freeze-drying (FD), pulsed vacuum drying (PVD), and hot-air drying (HAD). They found that IRD and HAD exhibited fastest drying along with maximum lipid oxidation. LIP analysis identified 1,541 lipid metabolites across 20 subclasses, with GPL being highest, followed by glycerides, GL, and SL. Besides reduction in lipid content, drying process also altered the structure of PE, PC, PS, PA, and LPC, which were involved in three major metabolic pathways: GPL, linoleic acid, and glycerolipid metabolism. Zhao et al. (2022) examined lipid oxidation in shrimp using LIP, identifying PE and PC as biomarkers linked to oxidation. They demonstrated that lipid species are highly susceptible to oxidation during drying, and the study emphasized the need to select drying methods that minimize oxidation to retain the nutritional and sensory properties of shrimp. Dry-cured meats undergo prolonged processing, during which lipid hydrolysis and oxidation play significant roles in flavour development. For example, TAG and PL get hydrolysed by lipase and phospholipase, respectively that release free fatty acid which subsequently undergo oxidation for the production of volatile compounds such as aliphatic hydrocarbon, responsible for meaty flavour (Guo et al., 2019). Guo et al. (2022) identified 581 lipid metabolites in dry-cured mutton ham using LIP, including glycerolipids, GPL, and SL as most abundant. The analysis revealed that the fermenting stage was critical, with significant downregulation of TAG such as TAG (18:1/18:2/18:3), PE (20:5/18:1), and TAG (16:1/18:1/18:4), and upregulation of metabolites like CE (20:3), FFA (24:6), LPC (20:3/0:0), and FFA (18:4). GPL and SL metabolism were the primary metabolic pathways involved in the curing process. Notably, FA level was raised while GP level was reduced after processing. Variations in the levels of key lipids like PC (12:0/22:2), PC (16:0/18:1), PE(P-16:0/20:4), and PS (18:0/22:6) were the primary drivers of changes in quality. Hence, the study indicated that the oxidation of free FA, particularly during the later stages of curing, significantly altered the flavour profile of the meat. However, in case of cocoa fermentation, no significant change in lipid composition composed of fatty acyl and glycerolipids (Herrera-Rocha et al., 2022). Similarly, Sirbu and Kuhnert (2021) reported no significant variation in these lipids during fermentation. However, during drying and roasting, some volatile fatty acids evaporate, and significant changes in TAG content occur. These findings suggest that cocoa fermentation does not substantially alter its lipidome, which remains stable throughout the process.

Some food products such as meat are prone to microbial contamination, posing a challenge for preservation. Irradiation has emerged as a non-thermal decontamination technique widely used in the food industry. Jia et al. (2021) applied irradiation to goat meat and used a quantitative LIP approach via LC-MS to study lipid composition changes. They identified 174 lipids from 12 subclasses, with increases in seven lipid species—TAG, PC, PE, LPE, Cer, LPC, and SPH—and decreases in five others—DG, PS, PG, PI, and SM. Notably, irradiation increased lipid content, particularly PUFA, improving the meat's preservation quality. In another study, Jia et al. (2022) applied natamycin, potassium sorbate, and sodium diacetate in preserving the quality of Hengshan goat meat sausage, identifying 70 characteristic lipids across nine subclasses using UHPLC-Q-Orbitrap. Li, Al-Dalali, et al. (2021) assessed the impact of spices on PL molecules during the processing of water-boiled salted duck (WSD) using Shotgun LIP. They identified 118 PL molecules and observed that the influence of spices on most of these molecules began during the first day of dry-ripening, gradually diminishing throughout the process.

Lipid oxidation is a major concern during food processing, leading to the formation of oxidation products that alter the flavour, texture, and nutritional quality of foods. Oxidative products such as hydroperoxides, conjugated dienes, and volatile compounds (e.g., aldehydes and ketones) are commonly detected in roasted and fried foods. The degree of lipid oxidation is directly affected by processing conditions like temperature and time. Roasting also increased colour, acid value (AV, oxidative stability, and radical scavenging activity, while fatty acid composition remained mostly unchanged (Zhang et al., 2020). AV and peroxide value (POV) are the key indicators of rancidity where AV indicates hydrolytic rancidity and POV reflect autoxidation. During roasting of macadamia nuts, minor changes in fatty acids, a slight decrease in triglycerides, but increased free fatty acids and peroxide values (Tu et al., 2021). Roasting significantly enhances the sensory quality, antioxidant content, and oxidative stability of macadamia nuts while maintaining overall lipid composition. Unsaturated TAG is particularly prone to enzymatic hydrolysis due to their lower melting points, making FFAs more susceptible to oxidation, resulted in the production of off-flavours and diminished nut quality. Additionally, lipid oxidation in various oils and nuts, revealing the degradation pathways of PUFA and their transformation into bioactive compounds such as oxylipins which assessed using MS-based LIP. Furthermore, oxidative products such as hydroperoxyoctadecadienoic acids (HPODEs) and oxylipins also contribute to health issues such as inflammation and metabolic disorders like Type 2 diabetes, obesity, and non-alcoholic fatty liver disease (NAFLD) (Misheva et al., 2022). Hence, determination of these compounds lead to alleviation of lipid oxidation as well as enhance the longevity of processed food products. In studies on rape bee pollen, LIP identified alterations in metabolic pathways related to GPL and linoleic acid metabolism during drying processes, indicating significant oxidative stress (Wang, Chen, et al., 2022c). Ferraz Teixeira et al. (2022) demonstrated the utility of UPLC-MS/MS for early detection of lipid oxidation in two infant milk formulas, finding an increase in oxylipins by day 7—specifically, ω-6 and ω-3 derived oxylipins in Formula 1 and DHA-derived oxylipins in Formula 2. Additionally, Ruesgas-Ramón et al. (2019) detected phytoprostanes, biomarkers of oxidative degradation, in coffee and cocoa by-products, with cocoa husk pods showing high abundance. Grüneis et al., 2019) also used the LC-MS/MS method to quantify epoxidized and hydroperoxidized triglycerides in canola oil, further illustrating the broad applicability of LIP in studying lipid oxidation in food matrices.

Hence, LIP acted as powerful tool in analyzing lipid composition as well as influence of food processing on lipid profiles. By identifying lipid oxidation products, key metabolic pathways, and bioactive lipids, LIP approaches help improve food processing techniques, enhance nutritional value, and ensure food safety.

## 7 Quality control and traceability

Food authenticity and traceability are key concerns for regulatory authorities worldwide, particularly for managing food safety. Food traceability not only provide food safety but also enhance product quality, competitiveness, and brand reputation for businesses (Carrera et al., 2024). LIP, offers detailed exploration of lipid profiles and the identification of lipid fingerprints in food raw products which is desirable for tracking its origin and characteristics. It played a significant role in authenticating oily fruits, nuts, oil, etc., targeted for adulteration due to their high economic value. Different researchers have implemented LIP technique to assess TAG profiles, fatty acid compositions, and tocopherol content as key classification parameters for judging the botanical and geographical origin of oily fruits, nuts, oil, etc. Campmajó et al. (2020) effectively distinguished adulterant level with error less than 6.1 % to classify ten nut types and detect adulteration of almond products with hazelnut or peanut using HPLC-FLD fingerprints with PLS-DA. Similarly, TAG composition analysis in hazelnuts, almonds, Tunisian peanut, and pecan nuts using LIP techniques such as HPLC-DAD, HPLC-RID, and LC-ESI-MS, highlighted its value in defining significant variation in the cultivar grown in different environment conditions and harvested at varying year. Rydlewski et al. (2020) demonstrated that ESI-MS lipid profiling effectively detected adulteration of avocado oil with soybean oil. Similarly, Huang et al. (2021) also identified lipid profiles in 13 peanut cultivars using UPLC-Q-TOF-MS, GC–MS, and OXITEST, and to identify lipid markers distinguishing high-oleic acid (OA) from non-high OA cultivars. Lipid profiles varied significantly among cultivars, and 11 lipid molecules were identified as potential markers for OA classification based on PLS-DA analysis. Guimarães et al. (2023) offered reliable tool for almond authenticity and geographical origin verification by developing a validated RP-HPLC-UV method to quantify 7 phenolic acids, 7 flavonoids, and tocopherols (α, β + γ) in 19 almond samples from the USA and Greece. The method showed excellent linearity (r^2^ > 0.99) and high recoveries, while chemometric analysis (PCA, AHC) revealed origin-specific bioactive profiles, and a decision tree identified a concentration-based marker for accurate origin prediction. Similarly, atmospheric solids analysis probe tandem mass spectrometry (ASAP-MS/MS) have revealed characteristic fingerprint of TAG in 20 different vegetable oils, including soybean, sunflower, sweet almond, and coconut oils, which aid in identifying the oil source in commercial applications (Pizzo et al., 2022). Furthermore, unique PL profiles have also been detected in nuts like walnuts, almonds, peanuts, cashews, pistachios, and pecans using HILIC-ESI-IT-TOF-MS (Song et al., 2018). These findings underscore the importance of PL profiling in food traceability, offering a robust method for ensuring food authenticity and origin of various food products. Moreover, untargeted LIP has been applied to differentiate truffle species, highlighting the potential of lipid profiling for food authenticity (Creydt & Fischer, 2022). Although lipid phenotyping in oily fruits and nuts remains limited, current research is promising, and LIP is expected to make significant contributions to the quality, authenticity, and traceability of agri-food products in the future.

Food adulteration and safety concerns have driven the development of traceability systems to authenticate raw materials, often requires different analytical techniques depending on the specific situation. For example, LC-HRMS have been employed to detect pork adulteration in beef. Thirty-five metabolite markers, including phosphocholines, carnitines, anserine, hypoxanthine, linoleic acid, and prolylleucine, were identified as key predictors (Windarish et al., 2022). Additionally, PLS and OPLS models predicted pork concentration in beef meatballs with high accuracy (R^2^ = 0.99). Similarly, TAG profiles obtained through DART-MS have been used to detect adulteration of soft cheese with vegetable oils, with detection limits less than 1%. Lipid profiling through various MS approaches, such as ESI-MS and LC-MS/MS, has also been effective in detecting fraud in products like avocado oil with soybean oil and distinguishing virgin olive oils from other varieties based on glycerophosphatidic acid and phosphatidylglyceride content (Criado-Navarro et al., 2019). Rysova et al. (2022) demonstrated that MALDI-TOF MS, particularly using low molecular weight spectra with lasso-regularized generalized linear models, can detect bovine milk adulteration in goat and sheep milk with prediction errors as low as 2.33–4.00% at low adulteration levels, though performance is limited by milk proteome complexity, protein degradation, and genetic variability. England et al. (2020) developed a rapid, solvent-free MALDI-TOF MS lipid fingerprinting method to discriminate bovine from non-dairy milks via simple dilution and matrix addition, offering a sensitive, inexpensive, and high-throughput approach for milk authenticity testing. Together, these studies highlight MALDI-TOF MS as a promising, cost-effective tool for detecting milk adulteration and verifying dairy authenticity with minimal sample preparation.

## 8 Applications of artificial intelligence in Understanding Lipid Profiling Data

**Fig. 2:**
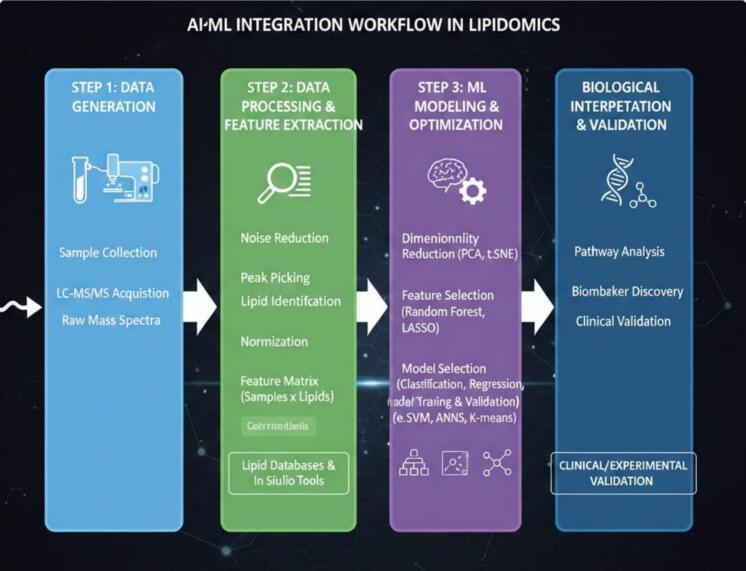
Artificial intelligence/machine learning workflow in lipidomics

The processing and quality evaluation of edible oils such as sunflower, palm, soybean, olive and flaxseed oils play a critical role in food technology, nutrition, and industrial product development. Conventional analytical techniques for testing composition, oxidative stability, and refining parameters of oil rely on analytical techniques such as **GC-MS**, **NMR**, **FTIR**, and LC-MS in the field of lipidomics. However, the integration and interpretation of oil quality data combined with regulatory texts, sensory evaluation, and literature pose significant data challenges. Fig. 2 represents the workflow of the AI/ML in lipidomics. In this context, **Large Language Models (LLMs)** like OpenAI's **GPT-4**, **SciBERT**, and domain-adapted transformers are emerging as powerful tools to support oil processing research and industry practices. These techniques offered innovative predictions for mining significant information from lipidomic related large volumes of scientific and technical literature. One of the vital applications of LLMs was in literature mining, where one can swiftly combine information across thousands of articles to identify known lipid biomarkers and understand processing-induced alterations in lipids or enhance knowledge of **methodologies by assembling various experiments for applications in food lipid analysis (**Xue et al., 2025). These models may also be used in characterization of lipids by aligning results of above mentioned conventional analytical techniques with established databases (eg. LIPID MAPS and HMDB), providing standardized classification and simplifying ontology mapping using resources like ChEBI and FoodOn (Witting et al., 2024). LLMs can be used to relate oil processing parameters to quality metrics (e.g., peroxide value, FFA, anisidine index etc). SciBERT, trained on scientific information, was mostly effective for mining literature on oxidative degradation, enzymatic processing, and solvent extraction (Beltagy et al., 2019). Osheter et al. (2023), presented a semi-autonomous, AI-powered sensing system for real-time assessment of edible oil oxidation using a low-field proton nuclear magnetic resonance (^1H LF-NMR) relaxation sensor. A large dataset of oils with varying oxidation levels was compiled, including raw T₂ relaxation curves. A convolutional neural network (CNN) was trained using supervised learning to classify the T₂ relaxation data into three oxidation categories i.e., non-oxidized, partially oxidized, and highly oxidized and the model achieved 95 % classification accuracy on unseen test data. Song et al. (2024), studied the effect of air frying on lipidomic fingerprinting of salmon was explored using ML-guided REIMS analysis and observed that ML method was able to predict significant variation in lipidomic fingerprinting of salmon. LLMs along with machine learning assisted in **prediction of shelf-life** or deterioration patterns based on oil composition and packaging variables (Addanki et al., 2022). Zhao et al. (2020) reported that combining ML with raman spectroscopy provide higher accuracy and faster detection of lipid quality. Furthermore, they indicated that random forest method provided highest and fastest test accuracy on detecting adulteration of soybean oil in olive oil (94.8% in 0.65 seconds) as well as adulteration of canola oil in avocado oil. Additionally, LLMs can function within analytical platforms as natural language interfaces, permitting operators to interact with datasets using natural language, for instance, querying the distribution of saturated fatty acids in processed foods or understanding oxidation developments under varied conditions of storage in different food products. LLMs may help in understanding experimental results by assimilating previous knowledge to conclude degradation pathways or recognize lipids signatures revealing various adulterants or the presence of non-affirmed oils. Another application may be for regulatory agencies, where LLMs can employed in mapping analytical data with nutritional, labelling and other standards demarcated by organizations like Codex Alimentarius, EFSA, FFSAI and the US FDA. This was allowed to spontaneously produce compliance documentation or recommend reformulations based on regulatory thresholds for trans fats and omega fatty acid ratios (Wu et al., 2025). In spite of many encouraging capabilities, LLMs need to be carefully used. Since the knowledge is dependent upon the superiority of their training data, and outputs required to be validated with domain expertise and empirical evidence. Though, as models become increasingly fine-tuned for scientific applications, predominantly when integrated with cheminformatics tools and curated lipidomics databases, they are poised to significantly boost lipid profiling workflows in food industry, connecting the gap between raw analytical data and meaningful interpretation.

## 9 Conclusion and future trends

Food lipids have historically been underexplored due to technical challenges, leaving much of their complexity unmapped. However, recent research highlights the critical roles lipids play in human health, including their functions as antioxidants and their influence on neural, immune, gastrointestinal, and cardiovascular systems. The growing field of LIP has begun to reveal the intricate lipid profiles of various foods, which are highly variable across species and sensitive to environmental factors such as growth conditions, maturity, and food processing. Food lipidomes offer insights into selecting resilient crop species, enhancing food production sustainability, and improving food traceability and safety. Current methods, mass spectrometry (MS)-based LIP, are limited by database availability and the complexity of food lipidomes. Future trends will likely include improvements in MS sensitivity and the creation of larger, more comprehensive lipid databases to facilitate the identification of lipid species and compounds. Furthermore, to fully realize the potential of food LIP, future efforts must focus on advancing analytical methods to overcome challenges posed by the complexity and diversity of lipids. Standardization of protocols, better understanding of the impact of food processing on lipid stability and bioavailability, and the development of large, comprehensive databases are critical next steps. Continued research in LIP phenotyping and lipid composition will contribute to the valorization of different food products and their by-products, supporting healthier diets and more sustainable food systems.

## CRediT authorship contribution statement

**Deepika Kathuria:** Writing – original draft, Validation, Conceptualization. **Sonal Aggarwal:** Writing – original draft, Data curation. **Akanksha Negi:** Software, Investigation, Data curation. **Riya Barthwal:** Writing – original draft, Software. **Aroma Joshi:** Visualization, Software. **Narpinder Singh:** Writing – review & editing, Supervision, Project administration.

## Funding declaration

NS received funding from SERB as JC Bose Fellowship grant and Bioversity International project, Grant No. L23DEL221

## Declaration of competing interest

The author is an Editorial Board Member/Editor-in-Chief/Associate Editor/Guest Editor for this journal and was not involved in the editorial review or the decision to publish this article. The authors declare the following financial interests/personal relationships which may be considered as potential competing interests: Dr. Narpinder Singh was guest editor for the special issue and he had no involvement in the peer-review of this article and has no access to information regarding its peer-review.

## Data Availability

No such data has been used in the study.
